# Induction of cervical disc degeneration and discogenic pain by low concentration *Propionibacterium acnes* infection: an in vivo animal study

**DOI:** 10.1186/s13075-024-03269-x

**Published:** 2024-01-31

**Authors:** Jie Li, Hui Li, Yilei Chen, Dikai Bei, Bao Huang, Kaifeng Gan, Peiming Sang, Junhui Liu, Zhi Shan, Jian Chen, Fengdong Zhao, Binhui Chen

**Affiliations:** 1 Department of Orthopaedic Surgery, Ningbo Medical Center Li Huili Hospital Affiliated to Ningbo University, 1111 Jiangnan Road, Ningbo, Zhejiang Province 315040 China; 2 Department of Orthopaedic Surgery, Sir Run Run Shaw Hospital, Zhejiang University School of Medicine, Hangzhou, Zhejiang China; 3 Key Laboratory of Musculoskeletal System Degeneration and Regeneration, Translational Research of Zhejiang Province, Hangzhou, Zhejiang China

**Keywords:** Cervical intervertebral disc, Intervertebral disc low virulent bacterial infection, Septic discitis, *S. aureus*, *P. acnes*

## Abstract

**Background:**

Although cervical intervertebral disc (IVD) degeneration is closely associated with neck pain, its cause remains unclear. In this study, an animal model of cervical disc degeneration and discogenic neck pain induced by a low concentration of *Propionibacterium acnes* (*P. acnes*-L) is investigated to explore the possible mechanisms of cervical discogenic pain.

**Methods:**

Cervical IVD degeneration and discitis was induced in 8-week-old male rats in C3–C6 IVDs through the anterior intervertebral puncture with intradiscal injections of low and high concentrations of *P. acnes* (*P. acnes*-L, *n* = 20 and *P. acnes*-H, *n* = 15) or *Staphylococcus aureus* (*S. aureus*, *n* = 15), compared to control (injection with PBS, *n* = 20). The structural changes in the cervical IVD using micro-CT, histological evaluation, and gene expression assays after MRI scans at 2 and 6 weeks post-modeling. The *P. acnes*-L induced IVD degeneration model was assessed for cervical spine MRI, histological degeneration, pain-like behaviors (guarding behavior and forepaw von Frey), nerve fiber growth in the IVD endplate region, and DRG TNF-α and CGRP.

**Results:**

IVD injection with *P. acnes*-L induced IVD degeneration with decreased IVD height and MRI T2 values. IVD injection with *P. acnes*-H and *S. aureus* both lead to discitis-like changes on T2-weighted MRI, trabecular bone remodeling on micro-CT, and osseous fusion after damage in the cartilage endplate adjacent to the injected IVD. Eventually, rats in the *P. acnes*-L group exhibited significant nociceptive hypersensitivity, nerve fiber ingrowth was observed in the IVD endplate region, inflammatory activity in the DRG was significantly increased compared to the control group, and the expression of the pain neurotransmitter CGRP was significantly upregulated.

**Conclusion:**

*P. acnes*-L was validated to induce cervical IVD degeneration and discogenic pain phenotype, while *P. acnes*-H induced was identified to resemble septic discitis comparable to those caused by *S. aureus* infection.

## Introduction

Neck pain is a prevalent global health issue, affecting a significant portion of the population worldwide. According to a comprehensive assessment of the global burden of lower back and neck pain, it is estimated that over one-third of people experience neck pain lasting for at least 3 months, highlighting the magnitude of the problem [1]. While various structures in the cervical spine can potentially contribute to neck pain as a result of degenerative changes, extensive research indicates that cervical disc degeneration leading to discogenic neck pain is the primary underlying cause [2].

The intervertebral disc (IVD), the largest non-neurogenic structure in the human body, typically lacks significant nerve fiber innervation in its outer annulus fibrosus region, which is normally not associated with pain sensation. However, when the IVD undergoes degeneration, it experiences structural and functional deterioration, triggering the activation and infiltration of immune cells [3]. This immune cell migration toward the IVD coincides with the emergence of nociceptive nerve fibers originating from the dorsal root ganglion (DRG) [4]. The DRG serves as a primary processing center for generating and transmitting pain signals, and in the case of disc degeneration, the IVD tissue exhibits a notable infiltration of immune cells. The inflammatory environment within the degenerated IVD tissue can consequently lead to heightened inflammatory activity within the DRG [5]. Simultaneously, the nucleus pulposus cells and infiltrating immune cells release cytokines and neurotrophic factors, inducing the expression of pain-related cation channels in the DRG [6]. The activated DRG can then release inflammatory cytokines and pain-related sensory neurotransmitters, thereby contributing to discogenic pain [7]. Thus, this pathological cascade elucidates the connection between IVD degeneration and the occurrence of neck pain.

In recent years, there has been growing evidence suggesting that intervertebral discs infected with low-virulent pathogens may contribute to disc degeneration and Modic changes of the endplate. This notion has been supported by numerous researchers [8–10]. Several studies have specifically highlighted the presence of *Propionibacterium acnes* (*P. acnes*) now also called *Cutibacterium acnes* (*C. acnes*), an anaerobic bacterium with low virulent, within degenerated lumbar and cervical intervertebral discs [11–13]. Initially, there was some debate regarding whether these findings indicated true infections or mere intraoperative contamination, as *P. acnes* is commonly found on the skin and is also known to cause infections associated with implants [14]. However, the accumulating evidence has reinforced the role of *P. acnes* in disc pathology. Recent research has revealed the presence of *Propionibacterium acnes* (*P. acnes*) in intervertebral discs prior to surgical tissue acquisition, suggesting a potential association between *P. acnes* and disc degeneration [13, 15]. While experimental models injecting *P. acnes* into the lumbar or caudal spine of animals have been reported to induce intervertebral disc or endplate degeneration, it remains unclear whether the outcomes in the cervical spine align with those observed in the lumbar spine due to anatomical variations and distinct adjacency [16, 17]. Consequently, further investigations are warranted to establish the consistency between cervical and lumbar spine results [18–20].

To date, no experimental studies have been conducted to explore the pathogenicity and consequences of *P. acnes* infection in the cervical intervertebral disc. Additionally, there is a lack of animal models specifically addressing bacteria-induced cervical disc degeneration and discogenic pain. Thus, the objective of this study was to investigate the impact of *P. acnes*, as well as highly virulent *Staphylococcus aureus* (*S. aureus*), on cervical disc degeneration in an animal model. Specifically, we aimed to assess the effects of low-concentration *P. acnes*-induced low-virulent infections in comparison with high-concentration *P. acnes* and *S. aureus*-induced septic discitis. Furthermore, we examined the behavioral responses of rats to mechanical and thermal stimuli, the growth of nociceptive nerve fibers in the disc’s endplate region, and the presence of pain and inflammation in the dorsal root ganglion (DRG) in the context of cervical disc degeneration induced by low-concentration *P. acnes* infection.

## Methods

### Animals

Seventy clean-grade male rats, with an age of approximately 8 weeks and an average body weight of 200 g, ranging from 190 to 210 g, were used in this study. Animals were provided by the Zhejiang University of Traditional Chinese Medicine Laboratory Animal Center. The animals were confirmed to be free of spinal disease by imaging preoperatively. All animals were operated following the ARRIVE guidelines and the UK Animals (Scientific Procedures) Act, 1986, and associated guidelines.

### Anterior cervical puncture

**Fig. 1:**
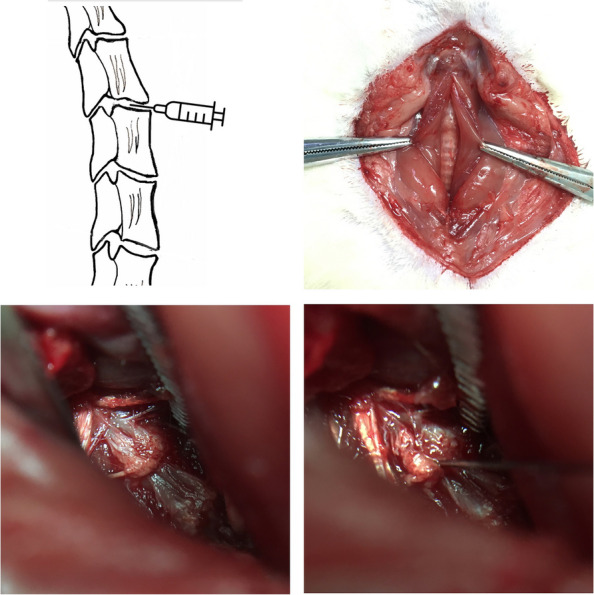
A blunt separation was made immediately to the right of the trachea toward the anterior cervical spine, and the tracheoesophageal sheath was pushed to the left and freed to the anterior vertebral space to expose the long cervical muscle

Eight-week-old male SD rats were anesthetized with pentobarbital sodium (30 mg/kg) by intraperitoneal push, and the experimental animals were placed in the supine position and fixed on the operating table. After aseptic skin preparation, a median longitudinal incision of approximately 2.5 cm was made in the front of the neck, the anterior cervical fascia was incised, and the sternocleidomastoid muscle was incised longitudinally in the direction of the muscle fibers to expose the trachea. A blunt separation was made immediately to the right of the trachea toward the anterior cervical spine, and the tracheoesophageal sheath was pushed to the left and freed to the anterior vertebral space to expose the long cervical muscle (Fig. 1A, B). The cervical longus muscle is pulled away to the sides to reveal the C3–C6 vertebral body and fully expose the intervertebral disc. The C3–C6 intervertebral discs were selected to complete the corresponding operations in each group. In the preoperative phase, cervical spine IVD levels were initially discerned through anterior–posterior X-ray images, with subsequent validation during surgery using an intraoperative C-arm. The fluoroscopy images from the C-arm revealed accurate needle punctures into the C3–4, or C4–5 IVDs in parallel through the anterior approach. The nucleus pulposus was punctured using a fine 29-G needle in an anterior-inferior to the posterior direction along the direction of the cervical disc (Fig. 1C, D). The entire operation was performed strictly aseptically, without the use of prophylactic antibiotics. After the operation, the skin was sutured, and rats were housed individually with free access to food and water.

### Experimental grouping

**Fig. 2:**
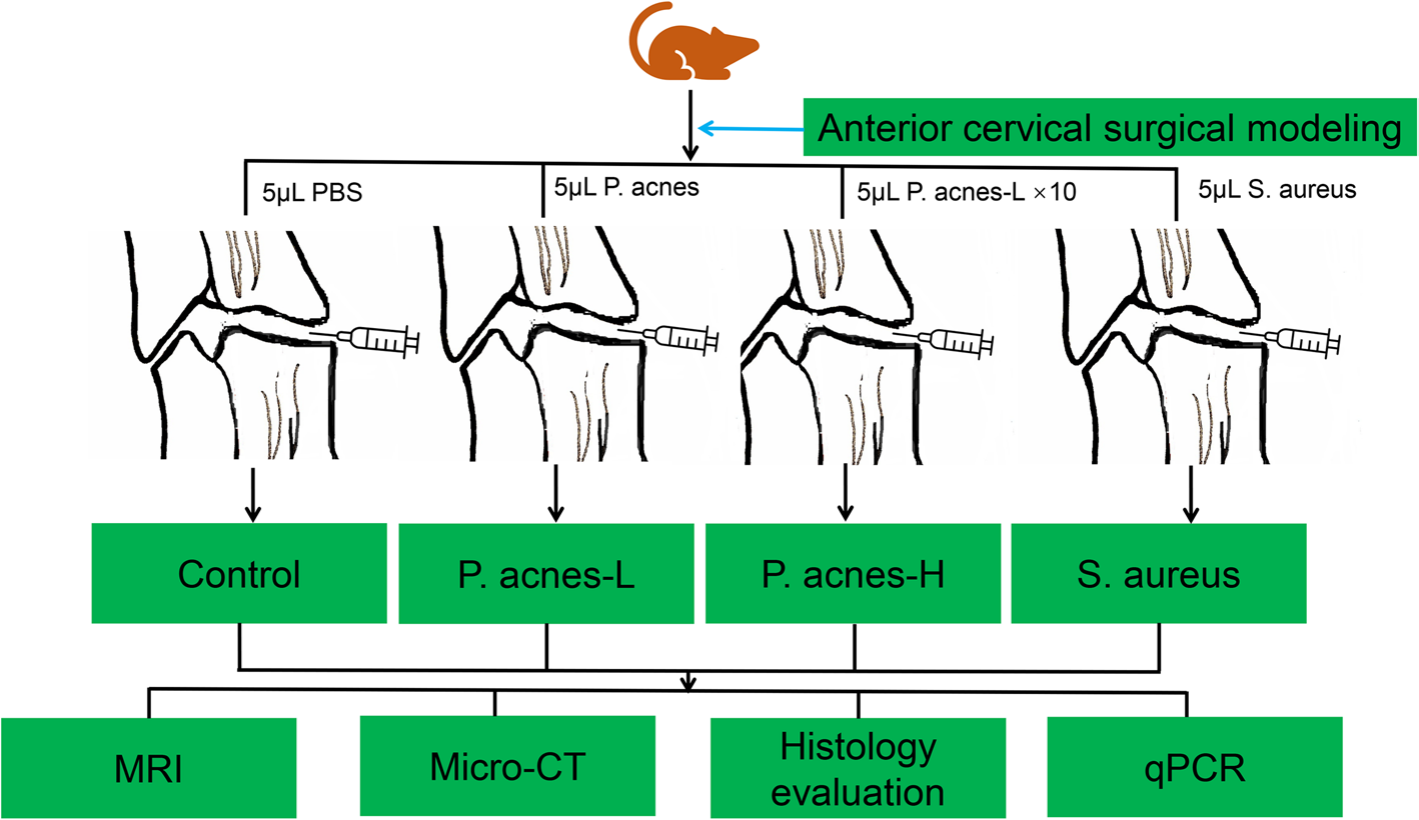
The animals were euthanized, and samples were collected to assess the various structural changes of the cervical intervertebral disc (IVD) in the modeled segments using MRI, micro-CT, histology, and qPCR

Seventy rats were randomly divided into a low- or high-concentration *P. acnes* (*P. acnes*-L, *P. acnes*-H) injection group, *Staphylococcus aureus*(*S. aureus*) injection group, or control group (20 in *P. acnes*-L and control group, 15 in *P. acnes*-H and *S. aureus* group). In the control animals, the C3–4 or C4–5 discs were injected with PBS (“control”). In the low-concentration *P. acne* rats, 5 μL *P. acne* (ATCC^#^6919 provided by Guangzhou Type Culture Collection, 1.6 × 10^8^ CFU/mL supported with PBS) was injected into the C3–4 or C4–5. In the high-concentration *P. acne rats*, 5 μL *P. acne* (ATCC^#^6919 provided by Guangzhou Type Culture Collection, 16 × 10^8^ CFU/mL supported with PBS). In *S. aureus* rats, 5μL *S. aureus* (ATCC^#^6538 provided by Guangzhou Huankai microbial, 1.6 × 10^8^ CFU/mL supported with PBS) was injected into the C3–4 or C4–5. The syringe needle may cause some damage to the cervical intervertebral disc. The control group distinguished the damage to the intervertebral space caused by the needle from the specific effects of bacterial infection. Following the modeling process, cervical MRI scans were conducted at 2 weeks (*n* = 7) and 6 weeks (*n* = 8) after the surgery. Subsequently, the animals were euthanized, and the samples were collected to assess the various structural changes of the cervical intervertebral disc (IVD) in the modeled segments using micro-CT, histology, and qPCR, respectively (Fig. 2).

### Animal complications condition and weight assessment

Intraoperative vascular and spinal cord injuries and the absence of postoperative complications such as incisional bleeding, ventricular rest, infection, limb paralysis, and eating disorders were evaluated. Weight changes at 2, 4, and 6 weeks postoperatively were recorded.

### Structural changes in the cervical IVD

#### MR imaging and evaluation

Cervical spine MRI was performed at 2 weeks and 6 weeks postoperatively, with a GE Sigma CV/I (3.0 T) using an animal surface coil to observe signal changes of cervical intervertebral disc on T2-weighted images. The parameters are as follows: scanning conditions for T2-weighted images in the sagittal plane were repetition/echo times of 2500/120 ms, a bandwidth of 31.25 Hz/Px, and an echo train length of 17. The scanning conditions for T1-weighted were repetition/echo times mini full of 550 ms, a bandwidth of 31.25 Hz/Px, and an echo train length of 2. A central sagittal image of the cervical spine was selected as a locating image for the next T2 FSE transverse-sectional scans at repetition/echo times of 3000/102 ms, a bandwidth of 31.25 Hz/Px, and an echo train length of 17. All imaging evaluations were performed independently by the authors and another spine surgeon, and disc changes were rated according to the Pfirrmann degenerative grading of the disc [21, 22]. When there was a controversy, the final rating was made after a consensus between the two.

#### Micro-CT scanning

After MRI examination at 2 and 6 weeks postoperatively, the animals were executed according to humane care, and the cervical specimens were isolated, placed in 4% paraformaldehyde for 24 h, and then transferred to 75% alcohol for further preservation. When micro-CT scanning, the specimen was fixed in a container with alginate glue and scanned at 25-µm layer thickness for the full length of the specimen. The scanning parameters were 70 kVp, 200 µA, 300 ms, a resolution of 1024 × 1024 (20 µm), and a field of view of 0.48 mm. One-millimeter thickness of vertebral cancellous bone above and below the modeled disc was selected using the micro-CT workstation software (µCT100, Scanco Medical AG, Bruttisellen, Switzerland), and trabecular bone parameters including bone volume fraction (BV/TV), trabecular thickness (Tb.Th), trabecular count (Tb.N), trabecular spacing (Tb.Sp), and bone mineral density (BMD) were measured and calculated.

#### Histological assessment

Following MRI scanning, the tissue samples underwent a series of steps for evaluation. Firstly, decalcification was carried out, followed by gradual dehydration, paraffin embedding, and sectioning into layers with a thickness of 5 μm. Subsequently, staining procedures including hematoxylin–eosin (HE) staining and Safranin O/Fast Green staining were performed. Once staining was complete, the morphology of the samples was examined using a light microscope. To evaluate the extent of cervical intervertebral disc degeneration (IVDs), the disc degeneration assessment scoring system was employed, which follows the methods previously described in the literature [15]. A higher score (ranging from 5 to 14) indicated a more severe degeneration of the IVDs.

#### Bacterial detection and quantitative polymerase chain reaction (qPCR) analysis

Cervical disc specimens were obtained, and genomic DNA was extracted from them. Subsequently, PCR was conducted using 16 s rDNA primers (Table 1), and the resulting DNA sequences were subjected to BLAST sequence alignment for identification of the corresponding bacterial community [13]. Separately, total RNA was extracted from the cervical disc specimens and purified using the RNeasy Mini Kit (Qiagen, Valencia, CA, USA). The purity and concentration of the extracted mRNA were assessed using a Nano-drop instrument (Biomed, USA) at wavelengths of 260 nm and 280 nm. Reverse transcription was performed at 45 ℃ for 50 min and 85 ℃ for 5 min using 5 × Prime Script RT Master Mix (Cwbio, Taizhou, China). The synthesized cDNA was then utilized for qPCR analysis of degeneration and inflammation-related genes, including Aggrecan, Col2a1, ADAMTS-5, MMP-3, TNF-α, and IL-1β, with β-actin serving as the internal reference. The qPCR reactions consisted of 2 µL cDNA, 10 µL Master Mix (Cwbio, Taizhou, China), 7 µL RNase Free, and 2 µL gene-specific forward and reverse PCR primers (synthesized by Sangon Biotech Co., Ltd., Shanghai, China) (Table 1). PCR amplification was performed using an ABI Prism 7500 system (Applied Biosystems, Foster City, CA, USA), starting with an activation step at 95 °C for 10 min, followed by 40 cycles of denaturation at 95 °C for 10 s, annealing at 60 °C for 20 s, and extension at 72 °C for 20 s. A final extension step at 72 °C for 1 min concluded the amplification process.

**Table 1:** Primer sequences

Rat gene	Primer sequences (5′–3′)	
Aggrecan	Forward	GCAGCACAGACACTTCAGGA
	Reverse	CCCACTTTCTACAGGCAAGC
Col2a1	Forward	GGCCAGGATGCCCGAAAATTA
	Reverse	ACCCCTCTCTCCCTTGTCAC
ADAMTS-5	Forward	AGTACAGTTTGCCTACCGCC
	Reverse	GATTTGCCGTTAGGTGGGCA
MMP-3	Forward	TTTGGCCGTCTCTTCCATCC
	Reverse	GCATCGATCTTCTGGACGGT
TNF-α	Forward	CATCCGTTCTCTACCCAGCC
	Reverse	AATTCTGAGCCCGGAGTTGG
IL-1β	Forward	AGGCTGACAGACCCCAAAAG
	Reverse	CTCCACGGGCAAGACATAGG
CGRP	Forward	CAGTGAAGAAGAAGCTCGCCTA
	Reverse	CAGTGTTGCAGGATCTCTTCTG
β-Actin	Forward	CTATGAGGGTTACGCGCTCC
	Reverse	ATGTCACGCACGATTTCCCT
*P. acnes* 16S rDNA	Forward	GGGTTGTAAACCGCTTTCGCCT
	Reverse	GGCACACCCATCTCTGAGCAC
*S. aureus* 16S rDNA	Forward	AGAGTTTGATYMTGGCTCAG
	Reverse	ACGGYTACCTTGTTACGACCT

### Evaluation of cervical discogenic pain

**Fig. 3:**
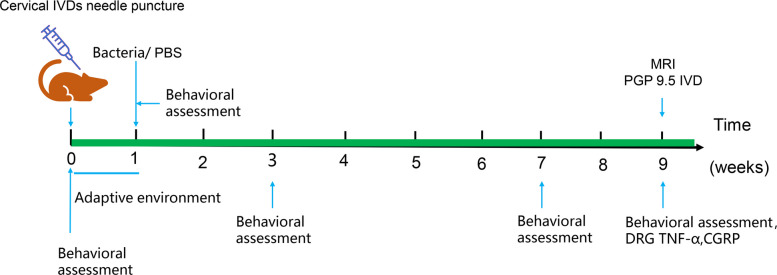
The rats were used for late cervical discogenic pain-related indexes, including nerve fiber ingrowth in the cervical disc endplate region, pain neurotransmitters in the DRG, and inflammatory factors in the DRG

Rats in the control (control, *n* = 5) and *P. acnes*-L (*P. acnes*-L, *n* = 5) groups continued to be fed under the same conditions as before until week 8 postoperatively. Behavioral assessments of rats in the control and *P. acnes*-L groups were completed at the beginning, preoperative, 2 weeks, and 6 weeks postoperatively to evaluate pain. The rats were used for late cervical discogenic pain-related indexes, including nerve fiber ingrowth in the cervical disc endplate region, pain neurotransmitters in the DRG, and inflammatory factors in the DRG (Fig. 3).

#### Behavioral assessments

To prevent assessment bias, the behavioral assessments were conducted by the same investigator who was blinded to the study group. As previously described, the mechanical sensitivity of the forepaw was evaluated using the Von Frey Mechanical Sting Test Kit, employing the elevation method. The steps involved in the assessment were as follows: Rats were initially acclimated in a separate box for 1 h. A series of Von Frey filaments (Aesthesio Precision Tactile Sensory Evaluator; DanMic Global LLC) were applied to assess the mechanical sensitivity of the forepaw. The filaments were applied in an incremental manner, starting from an initial force of 2.0 g and ranging from 0.4, 0.6, 1.0, 1.4, 2, 4, and 8 to 15 g. A positive reaction was determined if visible claw retraction or shaking occurred. If the response was positive, lower force filaments were applied, whereas if the response was negative, higher force filaments were used. The test comprised six individual stimuli, and the Von Frey force values were logarithmically transformed and averaged. Spontaneous guarding behavior was scored as follows: 0 for no guarding, 1 for mild removal of the body from the paw, 2 for uneven weight-bearing where some parts of the foot did not touch the ground, and 3 for paw licking with the foot fully raised. Prior to the Von Frey filament test, six observations were recorded, and the average scores were calculated. By following these procedures, the investigator ensured consistency and objectivity in the behavioral assessments.

#### Evaluation of nerve fiber growth in the cervical disc endplate region

To assess nerve fiber ingrowth within the cervical intervertebral disc, immunofluorescence detection of protein gene product 9.5 (PGP9.5) expression was performed. PGP9.5 is a marker for nerve fibers, and its presence indicates the presence of nerve fibers within the cervical intervertebral disc endplate region.

#### Assessment of cervical discogenic pain induced by *P. acnes*-L low virulent infection in rats

After 8 weeks postoperatively, the rats’ behavior was evaluated. Following this, cervical MRI scans were performed to confirm the presence of disc degeneration. Subsequently, the rats were euthanized using an overdose of sodium pentobarbital. Specimens of the cervical discs from the affected segments, as well as a portion of the dorsal root ganglion (DRG), were collected. The collected samples were subjected to fixation, decalcification, dehydration, paraffin embedding, and slicing. Additionally, another portion of the DRG was used for qPCR assay. The specific timeline and process for these procedures are outlined in Fig. 1. Immunofluorescence techniques were employed to detect nerve fibers in the cervical intervertebral disc endplate area. Furthermore, the expression of inflammation and pain-related neurotransmitters in the DRG was assessed using a combination of immunofluorescence, qPCR, and immunohistochemistry techniques.

### Statistical analysis

The data for all measurements are expressed as mean ± standard deviation. Statistical analysis was conducted using the SPSS 25.0 statistical software (IBM, USA). Continuous variables were analyzed using Student’s *t*-test or one-way analysis of variance (ANOVA), followed by the least significant difference (LSD) method for post hoc analysis of pairwise group differences. Ratios were compared using the chi-square test. *P* < 0.05 was considered statistically significant, indicating the presence of significant differences.

## Results

### Surgical complications and weight changes in rats

**Fig. 4:**
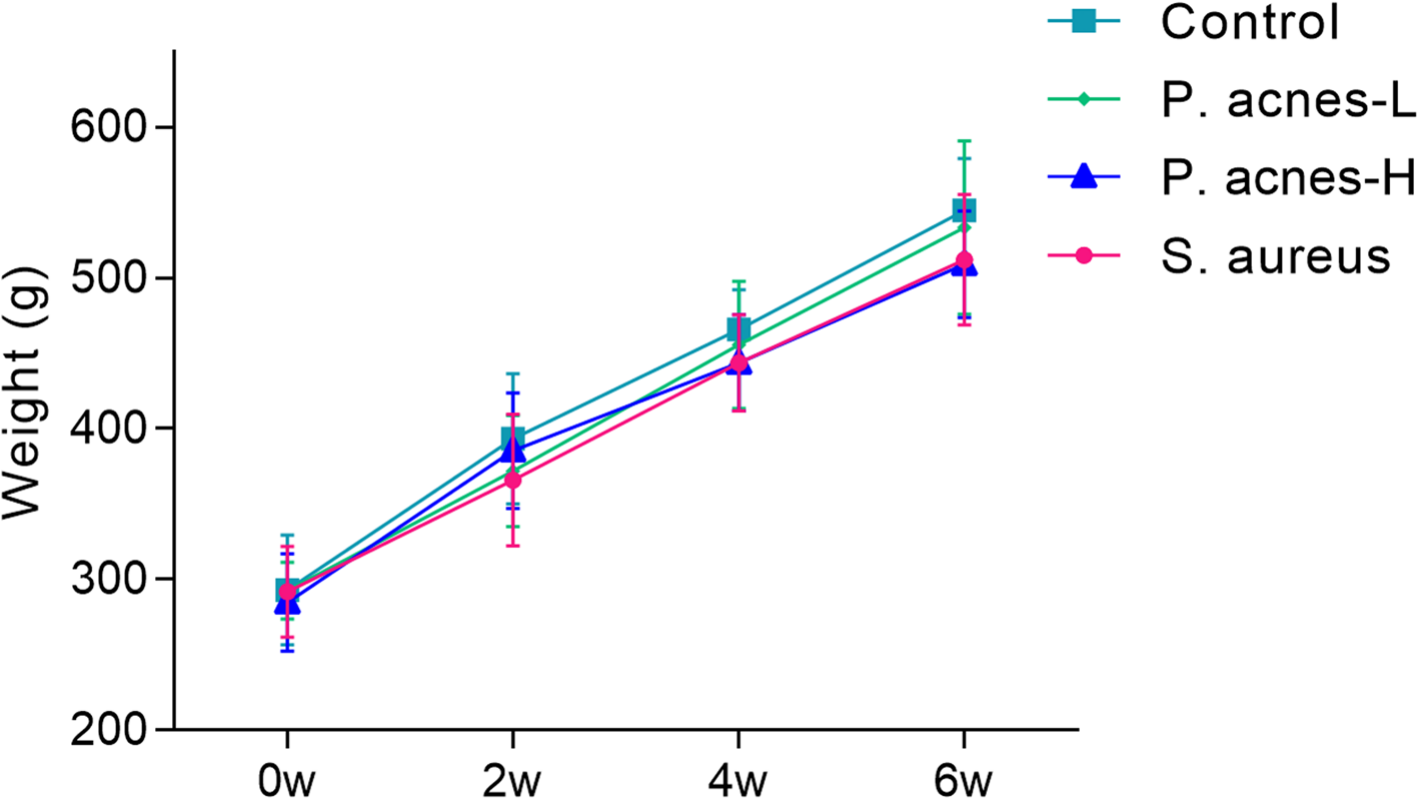
The body weights of rats in all groups exhibited a gradual increase after surgery, with no significant difference observed between the groups (*P* > 0.05)

The modeling process was successfully completed for all animals, and no intraoperative damage occurred to the blood vessels, respiratory tract, esophagus, or spinal cord. Furthermore, there were no instances of postoperative complications. The incisions achieved stage I healing approximately 1 week after surgery. Throughout the preoperative and postoperative observation periods, no unexpected deaths occurred. The body weights of rats in all groups exhibited a gradual increase after surgery, with no significant difference observed between the groups (*P* > 0.05) (Fig. 4).

### Differences in MRI findings between *P. acnes*-L-induced cervical disc degeneration and septic-like discitis

**Fig. 5:**
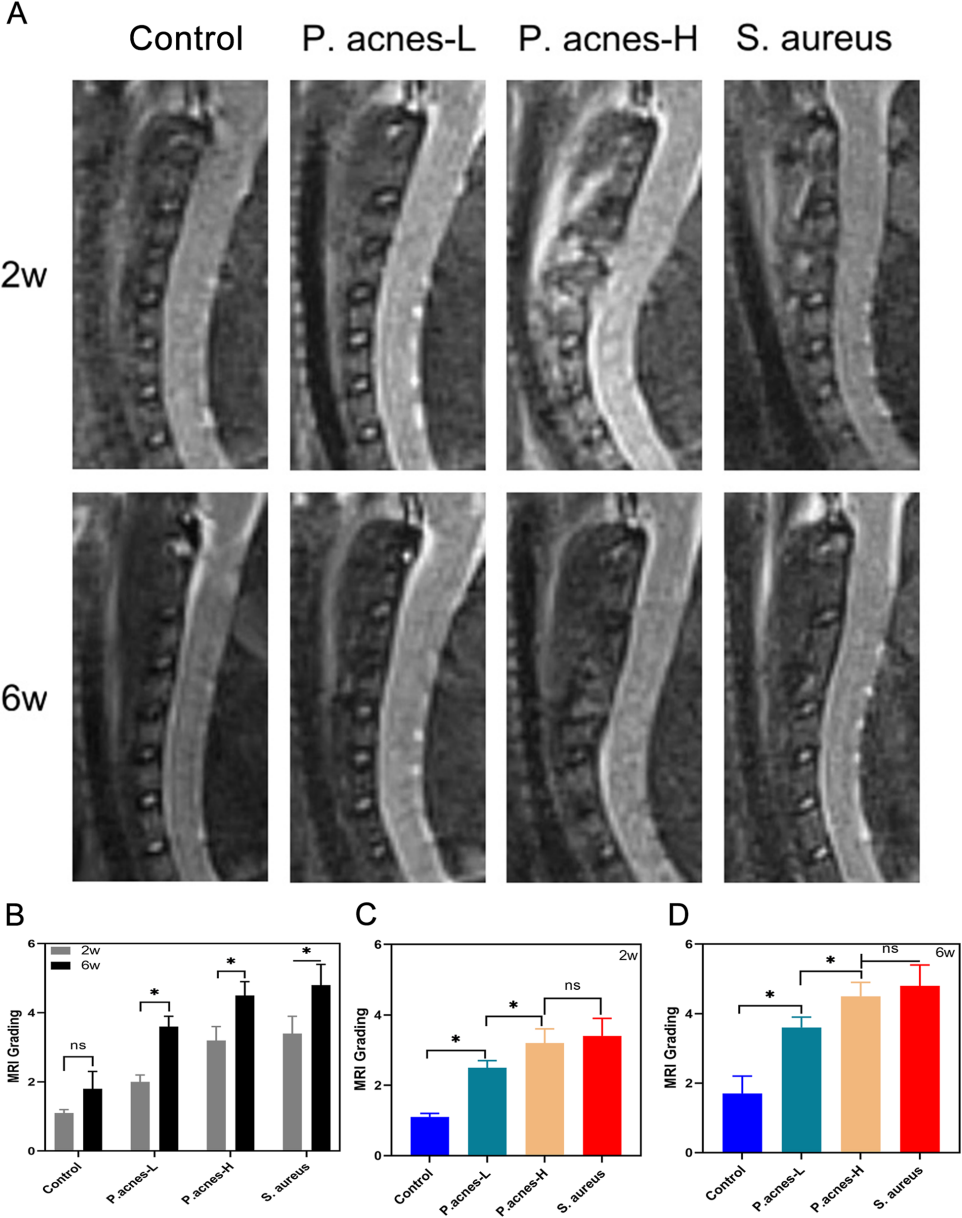
**A** Localized intervertebral space fusion. **B** The MRI scores of the modeled intervertebral disc segments at 2 and 6 weeks after surgery showed statistically significant differences (*P* < 0.05) in all groups except the control group. **C**, **D** Differences between the control group and *P. acnes*-L group were statistically significant (*P* < 0.05)

Two weeks after surgery, the intervertebral disc segments in the control group exhibited high signals on T2-weighted imaging (T2WI), indicating intact disc structures and normal heights. These signals were similar to those of normal intervertebral discs. In contrast, the *P. acnes*-L group showed reduced T2 signals, while the *P. acnes*-H and *S. aureus* groups displayed moderately mixed T2 signals. The latter groups exhibited evident loss of intervertebral disc heights, mixed signals, and unclear adjacent vertebral bone structures. Additionally, extensive high signals were observed in the prevertebral fascia, consistent with a typical case of septic discitis. Six weeks after the operation, the control group showed slightly reduced T2 signals in the intervertebral discs, indicating mild degeneration. In the *P. acnes*-L group, the intervertebral disc degeneration was further aggravated, with a significant reduction in disc volume, a further decrease in T2 signals, and blurred boundaries between the nucleus pulposus and the annulus fibrosus. This degeneration progressed toward a state known as the “black disc.” The *P. acnes*-H and *S. aureus* groups also exhibited reduced disc signals. In these groups, the signal of the prevertebral fascia returned to normal, and bone remodeling occurred in the intervertebral disc and vertebral bone structures after destruction. Localized intervertebral space fusion was observed (Fig. 5A). The MRI scores of the modeled intervertebral disc segments at 2 and 6 weeks after surgery showed statistically significant differences (*P* < 0.05) in all groups except for the control group (Fig. 5B). The differences in MRI scores between the *P. acnes*-H and *S. aureus* groups at 2 and 6 weeks postoperatively were not statistically significant compared to the control group (*P* > 0.05). However, the differences between the control group and *P. acnes*-L group were statistically significant (*P* < 0.05) (Fig. 5C, D).

### Differences in bone microstructure between *P. acnes*-L-induced cervical disc degeneration and septic discitis-like discs

**Fig. 6:**
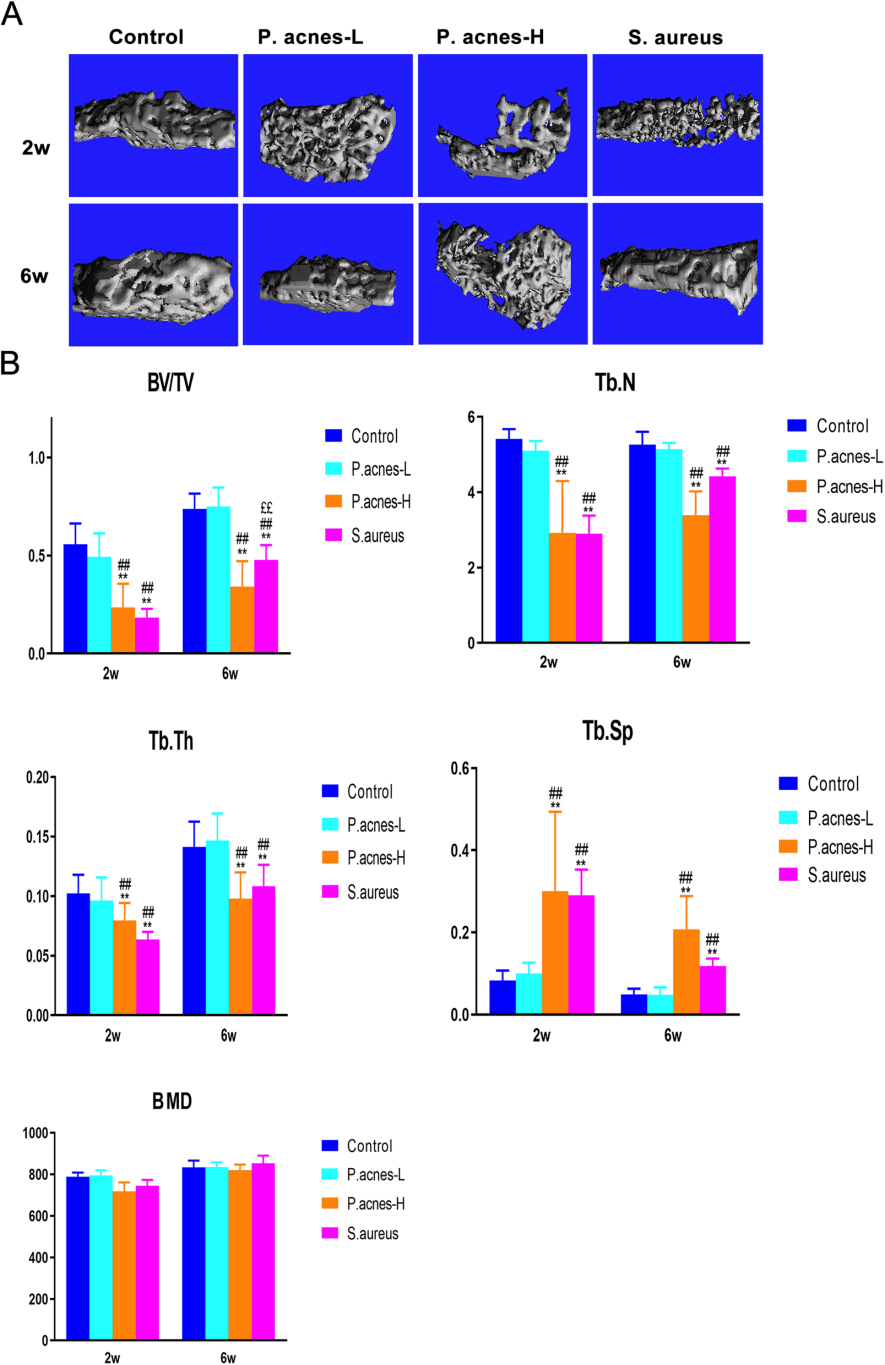
**A** Micro-CT 3D reconstruction of a typical image. **B** Bone microstructural analysis of the subchondral bone region of the endplates revealed that at 2 weeks and 6 weeks postoperatively. *Shows comparison with Control group, #shows comparison with P. acnes-L group, £shows comparison with P. acnes-H group

Typical images from micro-CT 3D reconstruction of the cancellous bone region of the vertebral body at 2 and 6 weeks postoperatively, above and below the intervertebral discs of the modeled segments in each group, at 1 mm thickness. Micro-CT 3D reconstruction of a typical image (Fig. 6A). At 2 weeks postoperatively, the bony endplates of the intervertebral discs were intact, and there was no destruction of the subchondral bone in the control and *P. acnes*-L groups, while the anterior aspect of the vertebral bodies in the *P. acnes*-L group showed a mild osteogenic reaction, and the anterior aspect of the vertebral bodies in the *P. acnes*-H and *S. aureus* groups showed a strong periosteal reaction, with the destruction of the disc structure and narrowing of the intervertebral space, the disappearance of the trabecular structure in localized areas, and the appearance of porosity or worm holing in the adjacent vertebral bodies. At 6 weeks postoperatively, the control group showed mild degenerative changes with no obvious changes in the vertebral bone; the *P. acnes*-L group showed obvious degenerative changes with narrowing of the intervertebral space, osteophytes at the margins of the vertebral body, and no obvious changes in the subchondral bone; and the periosteal reaction at the anterior aspect of the vertebral body of the modeled segments disappeared and was replaced by mature hyperplastic bone in the *P. acnes*-H and *S. aureus* groups, which were characterized by a strong periosteal reaction. replaced by mature hyperplastic bone. The intervertebral disc and bony endplate structures largely disappeared and were replaced by newly formed trabeculae connecting the upper and lower vertebrae, and bony fusion of the intervertebral spaces in the modeled segments occurred. Bone microstructural analysis of the subchondral bone region of the endplates revealed that at 2 weeks postoperatively, the *P. acnes*-L group showed a decrease in bone volume fraction (BV/TV), a decrease in trabecular counts (Tb.N) and trabecular thickness (Tb.Th), and an increase in trabecular spacing (Tb.Sp), compared with the control group, but the difference was not statistically significant (*P* > 0.05) (Fig. 6B). In the *P. acnes*-H and *S. aureus* groups, there was a significant decrease in BV/TV, Tb.N, and Tb.Th and a significant increase in Tb.Sp, with statistically significant differences (*P* < 0.01) when compared to the two groups; the rest of the parameters were not statistically significant, suggesting that significant osteogenic and proliferative repairs had occurred.

### Histologic differences between *P. acnes*-L-induced cervical disc degeneration and septic-like discitis

**Fig. 7:**
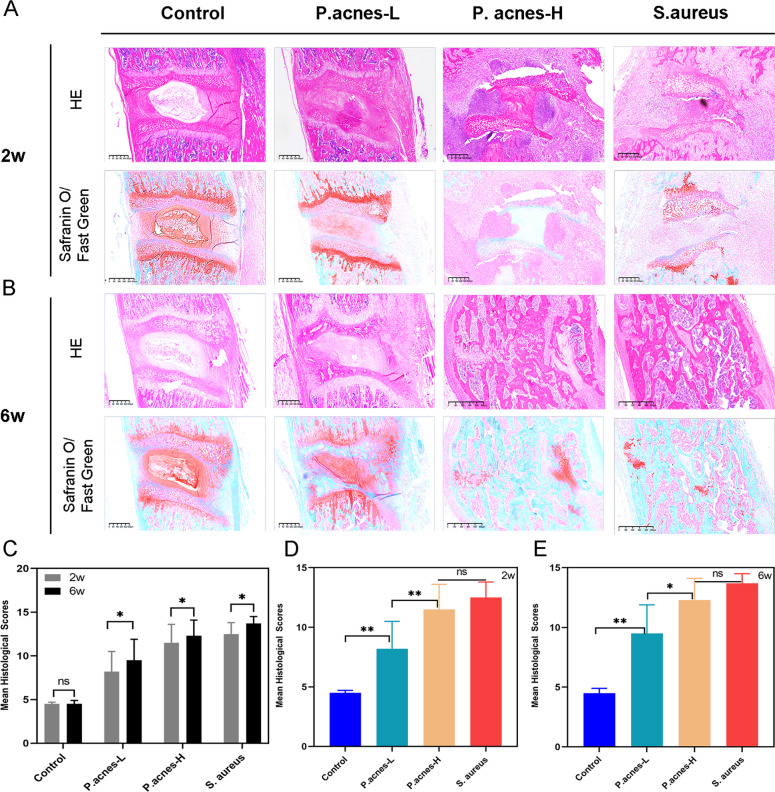
**A** The cell death in the central region of the intervertebral disc was complete in the *P. acnes*-H and *S. aureus* groups and was more severe in the *S. aureus* group. **B** At 6 weeks postoperatively, there was no obvious degeneration of the disc and endplate in the control group. **C**–**E** The histologic scores for each group at 2 and 6 weeks postoperatively

Central sagittal HE and red/green-stained sections of the modeled discs showed that at 2 weeks postoperatively, the control group had normal gross and cellular morphology. The control group showed normal gross and cellular morphology, whereas the P*. acnes*-L, *P. acnes*-H, and *S. aureus* groups showed nucleus pulposus cell death, nucleus pulposus dehydration, decreased disc height, and fibrous tissue proliferation. The cell death in the central region of the intervertebral disc was complete in the *P. acnes*-H and *S. aureus* groups and was more severe in the *S. aureus* group (Fig. 7A). The cartilage endplate structure was disturbed in both *P. acnes*-H and *S. aureus* groups, with chondrocyte proliferation and endplate osteoid. In both the *P. acnes*-H and *S. aureus* groups, the cartilage endplate structure was disturbed, with cartilage cell proliferation and endplate bone destruction, whereas in the control and *P. acnes*-L groups, the cartilage endplate structure was more intact (Fig. 7A). At 6 weeks postoperatively, there was no obvious degeneration of the disc and endplate in the control group (Fig. 7B). At 6 weeks postoperatively, the matrix in the *P. acnes*-L group showed further degenerative changes, ossification of the endplate region, and new bone formation at the vertebral body margin, whereas in the *P. acnes*-H and *S. aureus* groups, bony fusion of the intervertebral discs occurred, and the normal intervertebral disc structure was completely lost consistent with septic degeneration (Fig. 7B). In the *P. acnes*-H and *S. aureus* groups, the intervertebral discs underwent bony fusion with complete loss of normal intervertebral structure consistent with septic discitis (Fig. 7B). The histologic scores at 2 and 6 weeks for each group are shown in Fig. 7B. The histologic scores for each group at 2 and 6 weeks are shown in Fig. 7C–E.

### Differences in gene expression between *P. acnes*-L-induced cervical disc degeneration and septic-like discitis

**Fig. 8:**
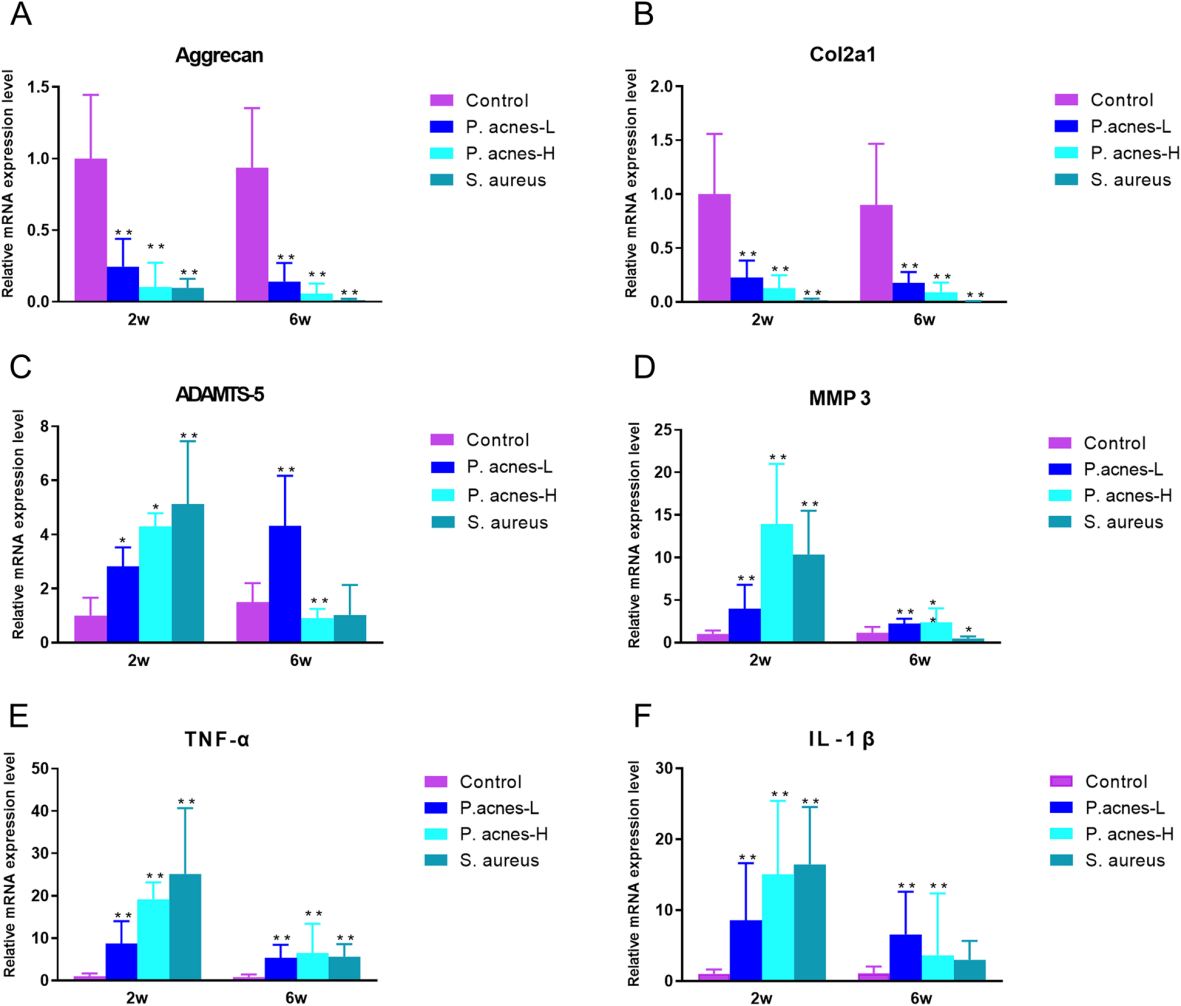
Quantitative PCR results of modeled segmental intervertebral discs at different time points. **A** Aggrecan, **B** Col2a1. **C** ADAMTS-5, **D** MMP3, **E** TNF-α, and **F** IL-1β

PCR analysis using 16S rDNA primers confirmed that the intervertebral discs (IVDs) of the modeled segments were free from bacterial contamination (Fig. 8). The qPCR results of target genes in the IVDs of the modeled segments at 2 and 6 weeks post-surgery are as follows: In terms of anabolism, the mRNA expression of Aggrecan and Col2a1 in the *P. acnes*-L, *P. acnes*-H, and *S. aureus* groups was significantly lower compared to the control group (*P* < 0.05). Regarding catabolism, at 2 weeks post-surgery, the expression of the ADAMTS-5 gene in the *P. acnes*-L, *P. acnes*-H, and *S. aureus* groups was significantly higher than that in the control group. At 6 weeks, the *P. acnes*-L group exhibited the highest expression of the ADAMTS-5 gene, which was significantly higher than the control group. The expression of the ADAMTS-5 gene in the *P. acnes*-H and *S. aureus* groups was significantly lower compared to the control group. The MMP-3 gene expression was highest in the *P. acnes*-H group, significantly higher than in the *P. acnes*-L and control groups. The expression was higher at 2 weeks compared to 6 weeks. Regarding pro-inflammatory factors, at 2 weeks post-surgery, the expression of TNF-α and IL-1β genes was significantly higher in the *P. acnes*-L, *P. acnes*-H, and *S. aureus* groups compared to the control group, with the highest expression observed in the *S. aureus* group. At 6 weeks, the expression of TNF-α and IL-1β genes decreased significantly in the *P. acnes*-H and *S. aureus* groups, but it remained significantly higher in the *P. acnes*-L group compared to the control group. These findings suggest that the *P. acnes*-L group, *P. acnes*-H group, and *S. aureus* group induced the expression of numerous inflammatory factors in the intervertebral discs. The high virulence of the *P. acnes*-H group and *S. aureus* group led to the release of a significant amount of inflammatory factors in the early stage, resulting in a robust but shorter-duration inflammatory response. In contrast, the *P. acnes*-L group induced a weaker but more persistent upregulation of inflammatory factor expression, similar to the control group.

### *P. acnes*-L-induced cervical discogenic pain

To evaluate nociceptive sensitization in the control and *P. acnes*-L groups following cervical discography, pain behaviors were assessed using spontaneous defensive behaviors and the Von Frey test at various preoperative and postoperative time points.

**Fig. 9:**
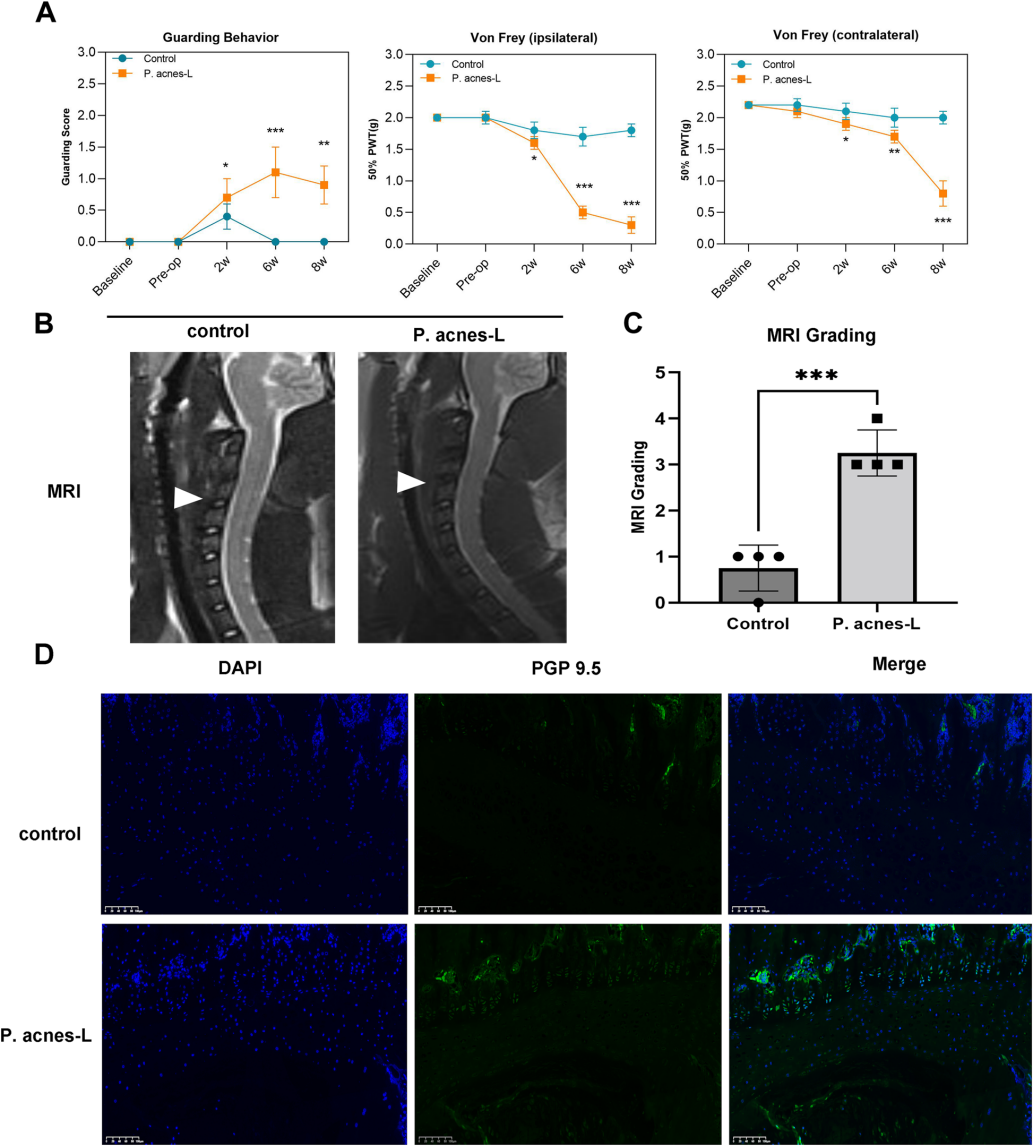
**A** The difference remained statistically significant compared to the control group. **B**, **C** The cervical intervertebral discs in the modeled segments exhibited additional degeneration at eight weeks postoperatively. **D** The staining revealed the distribution of the nerve fiber marker protein gene product 9.5 (PGP 9.5)

The results demonstrated an increase in defensive behavior in the *P. acnes*-L group at 2 weeks after the modeling procedure, followed by a decrease at 8 weeks postoperatively. However, the difference remained statistically significant compared to the control group (Fig. 9A). Furthermore, the ipsilateral Von Frey thresholds in the anterior paw began to decrease after the modeling surgery, coinciding with the onset of disc degeneration. These thresholds showed a significant decrease at 2 weeks and remained relatively low at 6 weeks. Subsequently, they further decreased and stabilized by 8 weeks postoperatively. The Von Frey thresholds in the contralateral anterior paw exhibited a relatively gradual change, also decreasing to a lower level by 8 weeks postoperatively and remaining stable. Notably, the Von Frey thresholds at all postoperative time points showed statistically significant differences between the *P. acnes*-L and control groups (*P* < 0.05) (Fig. 9A). These findings suggest that pain intensity increases as the degree of disc degeneration worsens. Furthermore, they indicate that the development of discogenic pain induced by cervical disc degeneration is gradual and persistent over time.

### *P. acnes*-L induces growth of nerve fibers into the endplate region of cervical intervertebral discs

At 8 weeks following anterior cervical spine modeling, immunofluorescence staining was performed on rat disc and adjacent vertebral body sections. The staining revealed the distribution of the nerve fiber marker protein gene product 9.5 (PGP 9.5), as illustrated in Fig. 9D. The cervical intervertebral discs in the modeled segments exhibited additional degeneration at 8 weeks postoperatively, as depicted in Fig. 9B, C. Simultaneously, in the *P. acnes*-L group, following low virulent infection of the cervical intervertebral disc, the number and density of nerve fibers significantly increased compared to the control group, as shown in Fig. 9D. This indicates that low-virulent *P. acnes*-L infection of the intervertebral disc resulted in cervical disc degeneration and promoted the growth of new nerve fibers within the endplate region of the cervical intervertebral disc.

### *P. acnes*-L induces the expression of pain neurotransmitters and inflammatory factors in cervical DRGs

**Fig. 10:**
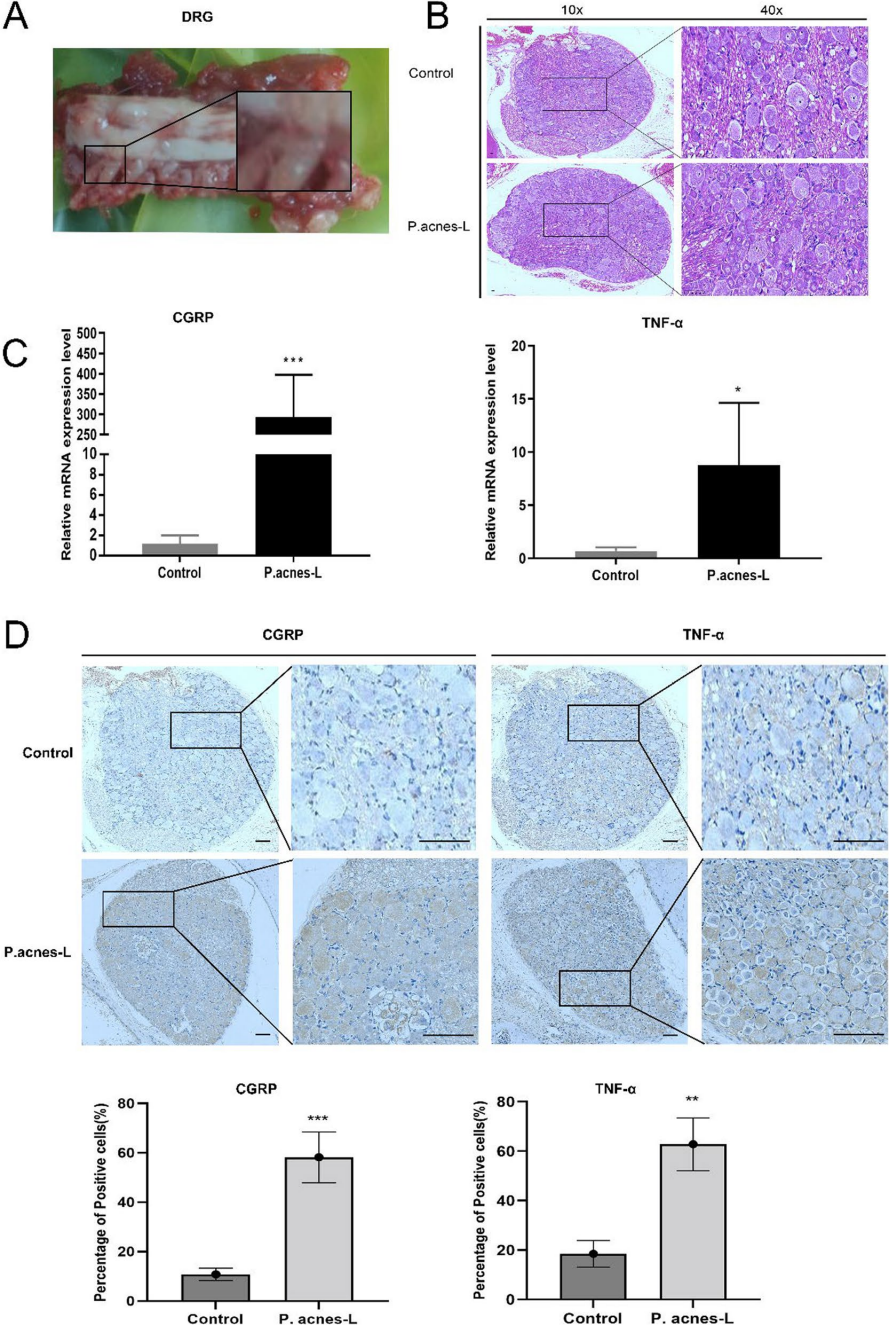
**A**, **B** DRG specimens from the modeled segments. **C** The quantitative PCR results showed a similar trend in TNF-α (*P* < 0.05) and CGRP (*P* < 0.005) expression in the DRG of the P. acnes-L group, aligning with the immunohistochemical findings. **D** Immunoreactivity of pain neurotransmitter CGRP (*P* < 0.005) and inflammatory factor TNF-α (*P* < 0.01) was notably increased in the stroma compared to the control group

**Fig. 11:**
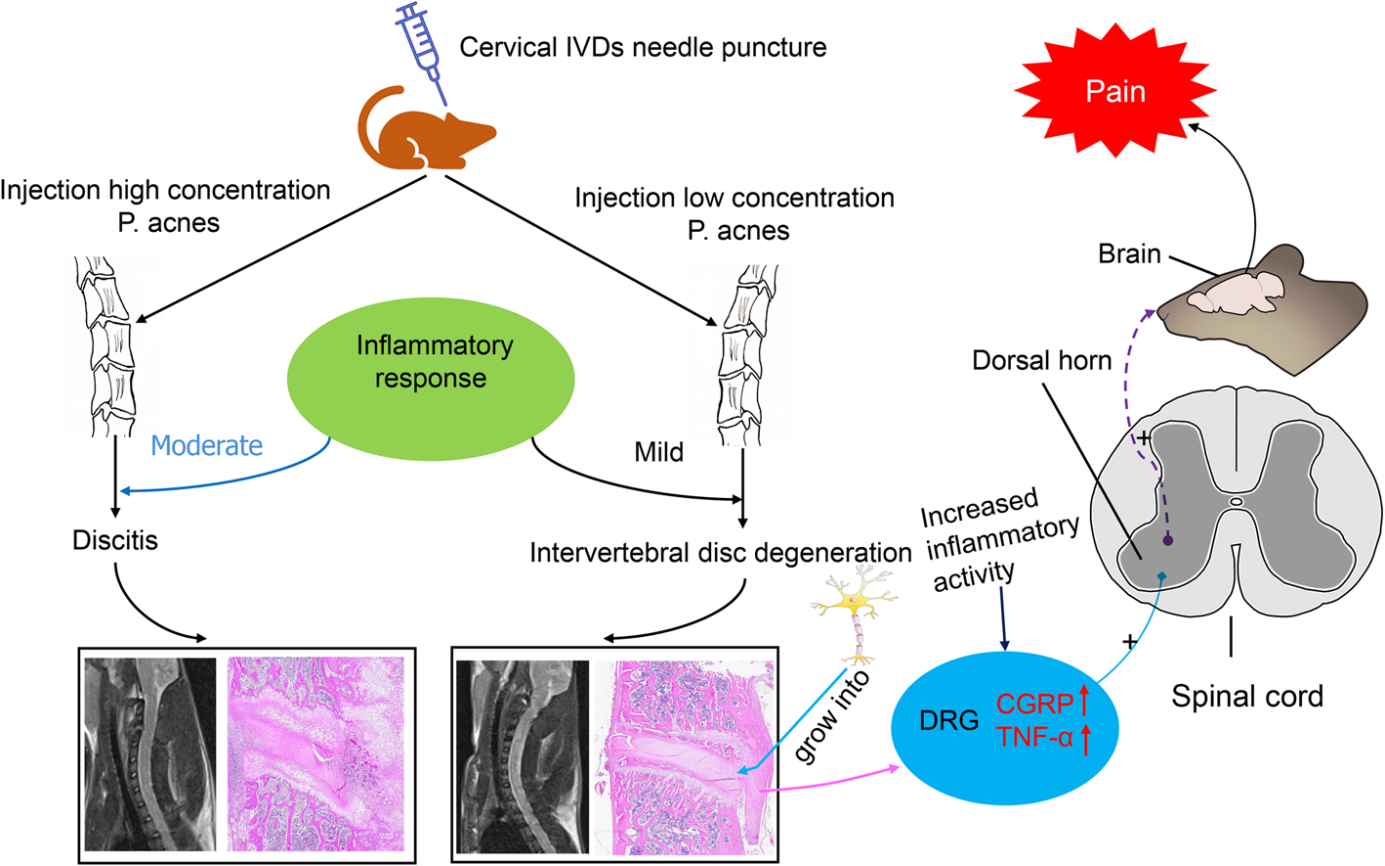
The expression of the pain-related neurotransmitter CGRP in the DRG increases due to inflammation stimulation, ultimately resulting in the generation of cervical discogenic pain

To investigate the impact of *P. acnes*-L low virulent infection on inflammation and pain stimulation in cervical dorsal root ganglia (DRGs), DRG specimens from the modeled segments (Fig. 10A) were collected at 8 weeks postoperatively for immunohistochemical staining analysis and PCR. Immunoreactivity of pain neurotransmitter CGRP (*P* < 0.005) and inflammatory factor TNF-α (*P* < 0.01) was notably increased in the stroma compared to the control group (Fig. 10D). The quantitative PCR results showed a similar trend in TNF-α (*P* < 0.05) and CGRP (*P* < 0.005) expression in the DRG of the *P. acnes*-L group, aligning with the immunohistochemical findings (Fig. 10C). These results indicate that *P. acnes*-L low virulent infection, following cervical disc degeneration, leads to heightened inflammatory activity in the DRG. Furthermore, the expression of the pain-related neurotransmitter CGRP in the DRG increases due to inflammation stimulation, ultimately resulting in the generation of cervical discogenic pain, as depicted in the specific pattern shown in (Fig. 11).

## Discussion

While various studies have confirmed the strong association between low virulent infection of *Propionibacterium acnes* (*P. acnes*) and intervertebral disc (IVD) degeneration, these investigations have predominantly focused on lumbar and caudal IVDs [6, 8, 18, 19]. It remains unclear whether low virulent infections of cervical IVDs exhibit similar manifestations and responses due to anatomical differences and their proximity to the spinal cord. Furthermore, it is yet to be determined whether *P. acnes* low virulent infection of the cervical IVDs leads to the induction of cervical discogenic pain and whether the structural changes resulting from *P. acnes* infection are concentration-dependent. Prior to the discovery of *P. acnes* infection causing degenerative changes in the IVD, various experimental models of IVD degeneration existed, with annulus fibrosus puncture/injury being one of the most classical and commonly used methods [23]. This indicates that puncture/injury is also a risk factor for IVD degeneration, but the extent of fibrous ring injury ultimately determines whether IVD degeneration occurs. In the literature, no statistically significant difference was reported in MRI scores of disc degeneration when one side of the annulus fibrosus was punctured using 29-G or larger gauge puncture needles compared to the control group [24]. Therefore, a 29-G puncture needle was selected for the injection of bacterial solution and PBS into the IVD in this study primarily to eliminate potential interference caused by the puncture needle itself during later assessments of structural changes in the IVD. Additionally, a close relationship between disc degeneration and pain has been established. The ability to establish an effective assessment method in animal experiments that accurately mimics human pain and allows for independent interventions is crucial for studying discogenic pain and inflammation associated with cervical disc degeneration. Equally important is the selection of a well-established animal model to serve as a control for low concentrations of *Propionibacterium acnes* (*P. acnes*-L) infection to explore the potential mechanisms by which *P. acnes* induces IVD degeneration through low virulent infection. In this study, we successfully developed a rat model of cervical disc degeneration and discogenic pain induced by a low concentration of *P. acnes*, which exhibited distinct characteristics from discoid discitis induced by a high concentration of *P. acnes* (*P. acnes*-H) but showed similarities to septic discitis induced by *Staphylococcus aureus* (*S. aureus*).

After modeling, the evaluation of body weight changes in rats revealed a gradual increase in body weight among all groups. The *S. aureus* and *P. acnes*-H groups showed relatively slower weight gains compared to others, but no statistically significant difference was observed in body weight gain between the groups. Monitoring recent weight changes is considered an indicator of the current nutritional status [25]. Previous research has indicated an association between inflammation and malnutrition, highlighting that severe systemic inflammation can impede protein synthesis, resulting in malnutrition and weight loss [26, 27]. However, Yang et al. [28] concluded that localized inflammatory response does not affect weight loss. Thus, based on the observed postoperative weight changes in this study, it was low thesized that *P. acnes*-L-infected cervical spine intervertebral discs (IVDs) may only generate a localized inflammatory response. Chen et al. [29] directly inoculated lumbar spine IVDs of rabbits with *S. aureus* and reported a mortality rate as high as 67%. In contrast, the present study injected high concentrations of *Propionibacterium acnes* (*P. acnes*-H) and *S. aureus* into the cervical spine IVDs of rats, but no mortality was observed. This discrepancy could be attributed to the relatively low virulence of *P. acnes* itself or the possibility that the doses of *P. acnes*-H and *S. aureus* used in this study were insufficient to trigger a systemic and intense inflammatory response. Consequently, the animal model employed in this study did not induce a systemic inflammatory response, thereby indicating its suitability for this research.

MRI serves as a highly sensitive diagnostic tool for identifying intervertebral disc (IVD) degeneration and discitis [30, 31]. The accuracy of MRI in diagnosing IVD degeneration is reported to be 100%, with a sensitivity of 96% for detecting infectious diseases. Furthermore, it demonstrates a specificity of 92% and an overall accuracy of 94% [31, 32]. When analyzing MRI images, IVD degeneration is identified by a reduction in signal intensity on T2-weighted images (T2WI), a decrease in disc height, and the presence of clefts within the IVD [33]. Ledermann et al. [32] suggested that septic discitis typically manifests as hyperenhanced signals related to the IVDs and vertebral bodies on T2WI, accompanied by lamina and vertebral body defects or erosions, with or without abscesses. In the present study, MRI scans revealed a progressive decrease in signal intensity on T2WI in the *P. acnes*-L group starting from 2 weeks after surgery. Similarly, the control group displayed a gradual decrease in signal intensity on T2WI from 2 weeks post-surgery. These MRI findings align with previous studies on disc degeneration, indicating that the cervical IVD in the modeled segment had undergone degenerative changes [34, 35]. Conversely, the MRI images of the *P. acnes*-L and *S. aureus* groups exhibited mixed hyperintensified signals, along with reduced volume of the IVD and adjacent vertebral structures in the modeled segment on T2WI. At 2 weeks postoperatively, an abscess was observed around the anterior aspect of the IVD, accompanied by erosion of the endplates and subchondral bone structures. Moreover, at 6 weeks postoperatively, the MRI images demonstrated a further decrease in T2 signal intensity and bony fusion of the affected IVD. These observations align with the typical presentation of septic discitis.

Histopathology serves as the definitive method for distinguishing between intervertebral disc (IVD) degeneration and septic discitis. Examination of histological samples using hematoxylin and eosin (HE) staining and saffron solid green staining revealed significant alterations in cell morphology, water content of the nucleus pulposus, disc height, and hyperplasia of vertebral fibrous tissues among the different IVD groups. Furthermore, notable differences were observed in the histological images of the *P. acnes* and *S. aureus* groups at varying concentrations.

The histopathological findings indicated that *P. acnes*-L infection in the IVD led to a localized and mild inflammatory response, which is consistent with the results reported by Białecka et al. [36]. In contrast, *P. acnes*-H and *S. aureus* infections in the IVD induced a pronounced and localized inflammatory reaction. This inflammatory response was characterized by early-stage destruction of the disc and subchondral bone structures, along with periosteal reaction in the anterior aspect of the vertebral body [37]. Subsequent stages demonstrated disc resorption, vertebral bone repair, and remodeling. These pathological manifestations are indicative of septic discitis and are clearly distinguishable from degenerative changes.

The quantitative PCR analysis indicated an inhibition of matrix synthesis in the nucleus pulposus and cartilage within the *P. acnes*-L, *P. acnes*-H, and *S. aureus* groups. ADAMTS-5 and MMP-3 were identified as key enzymes involved in matrix catabolism during different phases of disc degeneration induced by the respective bacterial infections [38]. The *P. acnes*-L group displayed consistent patterns observed in IVD degeneration studies, while the *P. acnes*-H and *S. aureus* groups exhibited more active degenerative processes [39]. Notably, all groups exhibited a substantial increase in pro-inflammatory factors, albeit with varying intensities and persistence. These findings suggest that factors beyond bacterial concentration and pathogenicity contribute to the development of clinical disc degeneration [39].

In this study, regardless of whether cervical intervertebral discs (IVDs) were inoculated with PBS, high or low concentrations of *P. acnes* or *S. aureus*, no significant impact on body weight gain was observed. Cervical MRI, micro-CT, histology, and quantitative PCR analyses were performed to investigate the effects of different inoculations on cervical IVDs. The results confirmed that injection of *P. acnes*-L into the cervical IVDs did not affect weight gain. However, it did induce degeneration of the cervical IVD and adjacent endplates. On the other hand, inoculation of *P. acnes*-H and *S. aureus* into the cervical IVDs resulted in the erosion of endplates and subchondral bone structures, along with bony fusion of the cervical IVDs. These structural changes align with the characteristic manifestations of cervical discitis. Furthermore, with the passage of time, *P. acnes*-L induction led to an exacerbation of cervical discogenic pain in addition to cervical IVD degeneration.

Under normal conditions, nerve fibers are typically limited to the outer layers of the annulus fibrosus (AF) in a healthy intervertebral disc (IVD). However, in pathological conditions like disc degeneration and chronic pain, sensory nerve fibers can extend into the inner layers of the disc [40]. Substance P (SP) and calcitonin gene-related peptide (CGRP) are sensory neurotransmitters associated with pain, and the presence of SP-immunopositive nerve fibers within the inner layer of the IVD has been observed in patients experiencing pain. Ohtori et al. [41] found that patients with painful discogenic pain exhibited a significantly higher number of PGP 9.5-immunopositive nerve fibers and TNF-immunopositive cells in endplates showing Modic type 1 or 2 changes on MRI compared to those with normal MRI signals. Zhang et al. [42] concluded that patients with severe back pain and reduced disc height displayed an increased density of sensory nerve fibers in the endplates, suggesting that the endplate could be a source of pain.

In the present study, rats in the *P. acnes*-L group exhibited prolonged mechanical nociceptive hypersensitivity after modeling, and this sensitivity intensified over time. Additionally, significant PGP 9.5-immunopositive nerve fibers were observed in the endplates of cervical IVDs at 8 weeks post-surgery in the *P. acnes*-L group, while almost no nerve fibers were present in the control group. These findings further support the notion that structural changes in the endplates of IVD may contribute to cervical discogenic pain. Therefore, it is suggested that structural alterations in the endplates following IVD degeneration could potentially serve as a source of cervical discogenic pain.

The results of the present study also showed that the expression of inflammatory factor TNF-α and pain neurotransmitter CGRP in the DRG was significantly upregulated 8 weeks after *P. acnes*-L infection with cervical IVD. The DRG is the main processing center for pain generation and transmission. Disc degeneration or herniation may lead to increased inflammatory activity in the DRG [43]. In a rat model of intervertebral disc degeneration model, nuclear factor κB and cyclooxygenase 2 (COX-2) expression levels were increased in the DRG on the left and/or right side of the disc [44]. In a rabbit model of torsional injury, after 60–90 days, it was observed that most DRGs expression values of neurotransmitters were significantly increased in most DRGs after 60–90 days [45]. In addition, activated DRGs can release inflammatory cytokines that affect the distal uninjured DRGs. The above results are generally consistent with the present study, which further suggests that low concentrations of *P. acnes* induced cervical discogenic pain on the basis of induced disc degeneration.

While this study successfully induced discogenic neck pain associated with cervical disc degeneration through the inoculation of *P. acnes*-L into the cervical intervertebral disc (IVD), there are still opportunities for further exploration of the underlying mechanisms of discogenic neck pain. Several limitations should be acknowledged. Firstly, the specific pathogenic mechanisms of *P. acnes* and *S. aureus* infections have not been thoroughly investigated, which hinders our understanding of the pathogenic substances and molecular processes involved in these infections. Consequently, the substantial differences in structural changes observed in the IVD caused by these bacteria and various concentrations of *P. acnes* remain unexplained. Secondly, this study focused primarily on evaluating imaging, histomorphometry, gene expression changes, tissue morphology, gene expression, and pain behavioral scores in animals. However, it did not delve into the molecular mechanisms of pain transmission or changes in brain function following pain stimulation. Understanding these submechanisms and examining alterations in functional brain regions after painful stimuli would provide a more comprehensive understanding of cervical discogenic pain. Furthermore, the study did not elucidate the molecular mechanisms underlying cervical discogenic pain induced by low concentrations of *P. acnes* in infected IVDs. Lastly, to determine the exact cutoff quantities, alternative methods such as enzyme-linked immunosorbent assay (ELISA) may be more appropriate. Building upon the foundation established by this animal model, future research should explore the molecular mechanisms underlying the transmission of cervical discogenic pain and investigate pain stimuli-induced changes in brain function. These investigations can deepen our understanding of the pathogenesis of cervical discogenic pain and potentially provide valuable insights for clinical treatment.

## Conclusion

Infection with low-virulent *P. acnes* results in degenerative changes in cervical discs, while *P. acnes*-H infection leads to symptoms resembling septic discitis comparable to those caused by *S. aureus* infection. In addition to causing disc degeneration, prolonged *P. acnes*-L infection promotes nerve fiber growth into the endplate region of the cervical disc, enhances inflammatory activity in the DRG, and triggers the release of the pain neurotransmitter CGRP, thereby contributing to cervical discogenic pain.

## Data Availability

No datasets were generated or analysed during the current study.

## Abbreviations

IVD: Intervertebral disc
*P. acnes*-L: Low concentration of *Propionibacterium acnes*
*P. acnes*-H: High concentration of *Propionibacterium acnes*
*S. aureus*: *Staphylococcus aureus*
qPCR: Quantitative polymerase chain reaction
MRI: Magnetic resonance imaging
DRG: Dorsal root ganglion
CGRP: Calcitonin gene-related peptide
PGP 9.5: Protein gene product 9.5
